# Insights from Drug Checking Programs: Practicing Bootstrap Public Health Whilst Tailoring to Local Drug User Needs

**DOI:** 10.3390/ijerph20115999

**Published:** 2023-05-30

**Authors:** Jeff Ondocsin, Daniel Ciccarone, Lissa Moran, Simon Outram, Dan Werb, Laura Thomas, Emily A. Arnold

**Affiliations:** 1 Center for AIDS Prevention Studies, Department of Medicine, University of California, San Francisco, CA 94143, USA; 2 Family & Community Medicine, Department of Medicine, University of California, San Francisco, CA 94143, USA; 3 Centre on Drug Policy Evaluation, St. Michael’s Hospital, Toronto, ON M5B 1W8, Canada; 4 Division of Infectious Diseases & Global Public Health, UC San Diego School of Medicine, University of California, San Diego, CA 92093, USA; 5 San Francisco AIDS Foundation, San Francisco, CA 94103, USA

**Keywords:** drug checking, harm reduction, community-based, substance use, fentanyl, North America

## Abstract

The year 2021 was the most deadly year for overdose deaths in the USA and Canada. The stress and social isolation stemming from the COVID-19 pandemic coupled with a flood of fentanyl into local drug markets created conditions in which people who use drugs were more susceptible to accidental overdose. Within territorial, state, and local policy communities, there have been longstanding efforts to reduce morbidity and mortality within this population; however, the current overdose crisis clearly indicates an urgent need for additional, easily accessible, and innovative services. Street-based drug testing programs allow individuals to learn the composition of their substances prior to use, averting unintended overdoses while also creating low threshold opportunities for individuals to connect to other harm reduction services, including substance use treatment programs. We sought to capture perspectives from service providers to document best practices around fielding community-based drug testing programs, including optimizing their position within a constellation of other harm reduction services to best serve local communities. We conducted 11 in-depth interviews from June to November 2022 via Zoom with harm reduction service providers to explore barriers and facilitators around the implementation of drug checking programs, the potential for integration with other health promotion services, and best practices for sustaining these programs, taking the local community and policy landscape into account. Interviews lasted 45–60 min and were recorded and transcribed. Thematic analysis was used to reduce the data, and transcripts were discussed by a team of trained analysts. Several key themes emerged from our interviews: (1) the instability of drug markets amid an inconsistent and dangerous drug supply; (2) implementing drug checking services in dynamic environments in response to the rapidly changing needs of local communities; (3) training and ongoing capacity building needed to create sustainable programs; and (4) the potential for integrating drug checking programs into other services. There are opportunities for this service to make a difference in overdose deaths as the contours of the drug market itself have changed over time, but a number of challenges remain to implement them effectively and sustain the service over time. Drug checking itself represents a paradox within the larger policy context, putting the sustainability of these programs at risk and challenging the potential to scale these programs as the overdose epidemic worsens.

## 1. Introduction

Drug checking at its most basic is a process whereby drug samples are analyzed to understand their chemical constituents and information about these substances is provided directly to the consumer. Drug checking services have existed for several decades [1,2], and in recent years, there has been a growing focus upon these services and their role in public health and harm reduction within and outside of North America [3,4,5,6,7,8,9]. The intended outcome of drug checking services frequently goes beyond providing a chemical analysis of drug samples with unknown compositions [1,10]. Some have argued that drug checking services may play a role beyond reducing drug-related deaths, including improved communication and risk management embedded in harm reduction practices [11]. Drug checking was initiated in the 1960s with psychedelic drugs in California [5]. In the 1990s, the emergence of the rave/festival scene in Europe and the associated ecstasy and amphetamine use led to concerns that non-injection drug users needed to have access to drug checking in order to reduce the likelihood of contaminants causing adverse health reactions or potential mortality [11,12,13,14,15,16]. Drug checking in the festival and event context has continued throughout the 2000s [17], and programs have emerged sporadically in North America over the last decade [5].

Overdose rates in both the USA and Canada have increased since 2000, with rates climbing faster since 2011 [18]. Overdose death rates, already high in the USA, have spiked since 2018 [19,20,21,22,23,24,25], and in 2021, more than 106,000 people died, with synthetic opioids involved in more than two-thirds of these deaths [25]. Overdose deaths in the USA have increased on an exponential growth curve since 1979 across a range of drug types [26], suggesting that overdose is contingent on a range of social, structural, and market factors that require more than drug-specific interventions [20]. The year 2020 was the first year since 1999 that overdose mortality in the USA was higher among Black individuals than White individuals [24], with overdose mortality among Blacks rising nearly 300% over this period compared to a 60% rise for non-Hispanic Whites [27]. Fentanyl has been present in Canada since at least 2012 [28], and by the mid-2010s Canada was experiencing a drastic rise in fentanyl-related deaths [18,29]. This trend has continued, with preliminary reports indicating that the first six months of 2022 saw 3556 opioid-related deaths, of which 76% involved fentanyl [30].

The introduction of fentanyl into the North American drug supply has raised the risk environment for people who use drugs (PWUDs) [31] due to its high potency by weight and covert presence [32]. Moreover, the illicit drug supply has become increasingly unpredictable with novel compounds, e.g., new fentanyl analogs, xylazine, nitazene-class opioids, illicit benzodiazepines, etc., emerging frequently. Counterfeit prescription pills containing fentanyl are a growing and concerning phenomenon [33]. Rising overdose-related mortality among PWUDs and vicissitudes in the drug supply [34] over the past decade have heightened the awareness among health service professionals, community leaders, and politicians of the need for service provision that will reduce the likelihood of overdose and other adverse drug-related outcomes. In concert with new service models, the notion of ‘harm reduction’ as a means to prevent or reduce the negative consequences of substance use has begun to be more widely accepted as a means to counter rising overdose and other drug-related health sequelae. This rising risk environment has fueled harm reduction innovations, especially drug checking. Drug checking technologies can confirm the presence of suspected drugs and common adulterants, as well as provide advanced warning of unexpected chemical contaminants. The most common and widespread form of drug checking is immunoassay test strips which provide yes/no information on the presence of single compounds (most frequently, fentanyl test strips [FTSs]) within a sample [19,35,36,37,38,39,40,41,42,43]. Other technologies include Raman spectrometers, used primarily by law enforcement for field testing presumed drug samples, and Fourier-transform infrared (FTIR) spectrometers, a technology that uses infrared light to scan samples in order to determine their chemical constituents [37]. Alternatively, various mass spectrometry technologies can provide quantitative information about drug samples, whether expected or unexpected [44,45,46], but are far more costly than other methods and less likely to be used in on-site drug checking [46]. FTSs are sensitive, fast, and are suitable for screening purposes [37]. Gas chromatography–mass spectrometry (GC-MS) is confirmatory and quantitative, but less immediate and has a greater cost, while FTIR is an intermediate technology. It is semi-quantitative, providing more information than an FTS and less than GC-MS but with a shorter timeframe [37,46].

Each of these forms of testing has related advantages and disadvantages with respect to analytical capabilities, cost of operation, time taken to test and provide feedback, and utility [37,44,46,47]. Given the variety of technologies in operation, it is not overly surprising that there is some discordance with respect to testing results [47]; however, the literature suggests that a greater level of detail provided incurs greater financial cost in terms of equipment and personnel to analyze and explain the findings. More granular detail may also be associated with increased response time, a factor which strongly militates against drug checking among structurally vulnerable populations [48]. Conversely, not having complete information reduces the utility of findings for people to make an *informed* decision about subsequent use of the checked drug [8].

Drug checking can occur both under supervision with a provider or *in situ* (i.e., FTSs) [49], where institutional contact with the user is minimal, potentially alleviating endemic mistrust of authorities among some while greatly reducing the likelihood that they will receive other drug-related or health-related services. However, immunoassay test strips are non-adaptive, i.e., FTSs will not provide information about the presence of nitazene-class opioids or xylazine. Mobile drug checking has the advantage of reaching more people but may be technically more challenging and costly [37,47,50]. Drug checking may provide additional channels to spread warnings about contaminants by word of mouth among communities of PWUDs [11,12,14,15]. Willingness to use services is complicated by social factors, including trust, stigma, and fears of police [48,50,51]. Research has also highlighted that people using drugs alone or in economically deprived conditions may have far fewer options to avoid risks post-test, e.g., they may not be in a position to not use drugs that have been confirmed to entail increased risk by drug checking analysis [41,48].

Drug checking programs remain controversial, not least due to the false perception that such programs encourage use [2,5,10,52]. It is notable how the recent opioid crisis has resulted in changing attitudes and practices, with greater policy and policing support for drug checking and associated harm reduction programs (such as supervised consumption sites) seen in many countries and within the USA [53,54,55,56]. However, in order to operate drug testing services, drug possession must either be decriminalized (which occurs rarely) or tolerated by authorities at least on a temporary basis. As such, drug checking programs and their staff exist in a complex space wherein testing is only possible as and when policy, policing, and public attitudes create legal cover for it to be carried out. Within the USA, the legal landscape for possessing drug testing equipment is dependent on individual jurisdictions, with varying degrees of legal ramifications for providers of such services where not officially sanctioned [3].

Studies exploring the benefits of integrating and co-locating services, such as healthcare provision and supervised consumption across harm reduction and outreach services, broadly may provide useful comparisons for drug checking integration [57,58,59,60,61]. The integration and co-location of services to manage the HIV and HCV epidemics among PWUDs have demonstrable benefits [59] and are robust enough to argue for a similar scaling up in the provision of integrated harm reduction and drug checking. However, some research has found that clients may only use sites for safe consumption but will not necessarily engage with other harm reduction services, or even know about their availability [58]. Additionally, the integration of supervised consumption within clinical care brings up a range of logistical issues, e.g., hours of operation, protection of personal identity and health information [58], and, potentially, contrasting approaches to substance use among project partners. For these reasons, the integration of services must be carefully developed to maximize benefits and minimize unintended barriers to the use of the primary service. Further research on service integration is urgently needed, especially in the context of integrated or co-located drug checking and harm reduction services in clinical settings.

Some research has examined stakeholder perspectives on implementing drug checking services in various settings [62], but little research has examined the perspectives of drug checking service providers about implementation experiences and best practices. In this study, we explore key themes emerging from the variety of services being provided in the USA and Canada. The issues described by drug checking service providers are illustrative of the heterogeneity of contexts within which drug testing is offered, the presumed objectives of such testing, and the degree to which testing can or does provide a focal point by which to offer health and other services under the umbrella of harm reduction. What we found is that local contexts, the politico-legal landscape, and the various technologies employed led to an extremely innovative and collaborative implementation of drug checking within a resource-constrained environment.

## 2. Materials and Methods

Two trained qualitative interviewers (D.C. and L.M.), one of whom is a clinician, conducted 11 in-depth interviews from June to November 2022 via Zoom with harm reduction service providers, clinicians, researchers, and drug checking lab technicians to explore barriers and facilitators around the implementation of drug checking programs. Collaboration with the “Ending the Epidemics Coalition”, a network of providers and advocates working to address HIV, Hepatitis C, STIs, and overdose in California, initially identified several key informants based in California. Other key informants were directly recruited because of current or past involvement in a geographically diverse array of drug checking programs in the USA or Canada and included published experts who are well known in the field. In some cases, our team used snowball sampling to invite specific key informants who could offer particular insights into areas that our team needed additional information on, such as integrating drug checking programs into clinical care settings or combining FTSs with spectrometry-based drug checking technologies. All participants were provided with a pseudonym for anonymity.

Interview topics included insights from implementing and running drug checking programs, logistical and political hurdles in initiating drug checking, ability to scale up existing programs, potential for integrating drug checking with other health promotion services, and best practices for sustaining these programs taking the local community and policy landscape into account. Interviews lasted 45–60 min and were recorded and transcribed. The Institutional Review Board at the University of California San Francisco reviewed and approved our study protocol, and participants provided verbal consent prior to interview activities.

A small team of seasoned analysts (E.A.A., L.M., S.O., and J.O.) read through the entire dataset and met to discuss and identify key thematic areas for additional exploration and systematic analysis. Initial thematic categories were based on the interview guide and were developed through team discussions. Using the tenets of thematic analysis [63], each analyst then reduced the data across a set of transcripts and organized key narrative passages into more detailed analytic memos, exploring patterns across the interviews themselves. This process identified several notable themes, including the instability of drug markets, various novel strategies to develop and support drug checking services, necessary training and capacity building to improve drug checking rollout and availability, and the promise and peril of integrating drug checking within other services. The team met regularly throughout this process, discussing and delving into the data to establish refined understandings for each thematic area. These refined versions of each analytic memo provided the backbone of the results presented below.

## 3. Results

Participants in this study identified drug checking as a necessary intervention due to ongoing instability and the introduction of potent synthetic drugs into the North American drug supply. They further recognized that existing technology is adaptable to current needs, but that scaling this intervention in order to achieve its goals requires substantial investment in staff training and the introduction of robust policy changes. Finally, participants indicated that there was significant potential to thoughtfully integrate drug checking services into existing networks of care, including within some healthcare settings. Eight participants were USA-based, two were Canada-based, and one worked in both the USA and Canada. Two participants were clinicians, four were academic researchers, and five were frontline service providers, including people currently and formerly involved with drug checking.

### 3.1. Instability of Drug Markets

The emergence of fentanyl in the USA and Canada has had a substantial impact on drug markets in these countries and has driven the overdose crisis to dizzying heights. Jason, who worked in drug checking in Vancouver, reported on what is believed to be the first fentanyl overdose at a supervised consumption site in Vancouver, setting the stage for the establishment of drug checking as a vital service in the city:

*“Someone brought this purple, pebbly substance into Insite and used it at one of the booths and overdosed. And all of a sudden word spread really fast that there was this purple substance going around that was dropping people left, right, and center. And no one really knew what it was, I don’t think, at the time necessarily that it was fentanyl.”*

*(Jason, drug checking technician)*

Participants reflected on these changes, highlighting how fentanyl emerged on the scene, first adulterating existing street opioid supplies before later emerging as a distinctly separate product (although this has not been true of all USA and Canadian locations reported on in this study). Additionally, growing availability of stimulant-type drugs, such as methamphetamine and counterfeit prescription medications containing fentanyl, are also likely contributors to overdose risk, broadening the potential reach of drug checking.

#### 3.1.1. Inconsistent Drug Supply

Changes to the drug market did not occur in the same fashion across these disparate regions, and drug market instability was mentioned by both American and Canadian participants. Eli in North Carolina, who runs a mail-based drug checking service receiving samples from within and outside North Carolina, found that even within samples sourced from the same place on the same day, components of the drug supply varied widely, almost idiosyncratically:

*“When I started doing the drug checking work on the ground in North Carolina, I was like, “Okay. We’re going to do geographic sampling. We’re going to get mountains. We’re going to get the coast. We’re going to cover the whole state.” And I thought that that was the right epidemiologic sampling frame to understand the drug supply. What we find on a daily basis is that the drug supply in one place will be changing bag-to-bag, hour-to-hour, person-to-person, and there is no consistency.”*

*(Eli, researcher)*

Issues with inconsistent supply were not limited to the USA. William, a researcher based in the USA and Canada, found, while quantifying drug concentrations in Canadian samples, a wide variability in fentanyl concentration that likely contributed to rising overdoses: “And what’s remarkable is that it varies from, if you look at February 2021, a high of 6.7% to-I think this is the low here of June 2021, so just three months later-to 2%.”

As Eli noted, downward pressures influence the illicit supply—“There’s been a lot of interdiction pressures around the globe that have led to the kind of black market needing to have a product that is more compact in packaging and more potent so that they can transfer those across borders or across places more easily”—resulting in stronger and more concentrated products. This has created a greater need for drug checking technologies to stay abreast of a drug supply with greater potency and the introduction of unknown products that may further drive overdose risk and cause other health sequelae.

#### 3.1.2. Chasing the Next Contaminant

Instability has long been a hallmark of the North American drug supply, but new contaminants and additives have been emerging at shocking rates in recent years, as Eli describes: “*the street supply is now more treacherous and unpredictable than anything that I’ve ever seen in the last 20 years. I’ve been doing this work*.” Participants, including Michael, a clinician, highlighted issues of scale in measuring the contamination problem, where among people who use opioids daily, fentanyl is now presumed to be ubiquitous in the supply: “*I think for people who have opiate use disorder, the assumption is that everything just has fentanyl and that that’s the way to go about it*.” However, among those not using opioids, or using counterfeit pressed pills with unknown ingredients, he notes that the issue of contamination can be immensely dangerous: “*I think it’s rare events, but I think those rare events are quite catastrophic and… and I don’t think it’s actually that common, but the thing is that when it happens once, it will equal a death*.” Amid ongoing reports of fentanyl adulteration of the stimulant supply, some participants thought these were largely overblown and that accidental contamination may be more related to elements of the selling and trans-shipment of drugs:

*“There’s been this narrative around that dealers are tainting the stimulant supply in a way to make it more addictive to people and to have them come back or whatever […] I think there’s two probably more prominent reasons why this is happening. One, I think it…there’s just common switch-ups at the point of sale. You have your little bag, you have your little fanny pack with your different things that you’re selling, and you have white powders which one could be cocaine, which one could be fentanyl, and one could be methamphetamine, and you give the…the wrong white baggie to the wrong person. […] I think a lot of these substances are either trafficked together or…or kind of put into baggies in–in the same place, and I think there’s probably cross-contamination at those sites.”*

*(Michael)*

Whether contamination is accidental or intentional, drug checking technologies offer the promise of providing vital information about the presence of new contaminants in the drug supply, such as the veterinary sedative xylazine and high-potency non-fentanyl opioids, such as nitazenes, in the opioid supply.

### 3.2. Drug Checking Services in a Dynamic Context: Constraints and Innovations

#### 3.2.1. Technology

Nearly every participant commented on the lack of a single existing ideal technology for drug checking, as well as the variety of machines and devices that are being used in North America to provide this service. Fentanyl testing strips (FTSs) are relatively inexpensive to produce, easily distributed, and can be used directly by clients to test their drugs. However, they are limited in terms of information provided, user error is common, and among people who regularly use fentanyl or opioids where fentanyl is commonly presumed present, their utility is low. High rates of false positives or negatives made some interviewees cautious about the use of FTSs, while allowing that FTSs provided valuable evidence to other drug checking modalities due to their high sensitivity.

By way of contrast, spectroscopy-including FTIR [Fourier-transform infrared spectroscopy] technology provides a greater level of detail and range of drug testing possibilities, albeit with lower sensitivity. However, such technology requires greater initial investment, is difficult to transport, and is intended to be used in controlled conditions, rather than at the point of use or on the street. These machines are also subject to the limitations of their chemical libraries, which can lead to some substances being overlooked, particularly in the absence of other modes of confirmatory testing. Spectroscopy can detect absolute and relative concentrations of a wide variety of chemicals in a sample, but there remain limits to even this form of technology with respect to detecting fentanyl (or associated compounds) at low levels. FTIR can generally not detect opioids that constitute <5% of a sample [37], while in real-world conditions, the detection limit may be closer to 10% [64], levels that may result in overdose.

While limitations are evident, it also important to highlight that the enterprise is not considered futile in terms of the available technology:

*“If I had to start from scratch to make a perfect machine for point-of-care drug checking, I would probably end up with FTIR. It is fast. Samples can be run in less than ten minutes. You get the results right back to the individual. You don’t have to destroy the drugs … It’s a great technology. And the other cool thing I think about it as opposed to the lab-based work is that we were able to train people with zero chemistry background to use it with high fidelity.”*

*(Eli)*

FTIR, although not ideal and challenging for people who need to use in the moment, can be used after the fact, yielding important information about the drug supply that can be provided to the community in the form of reports or alerts about potential contaminants encountered in specific drugs or across geographic areas:

*“They [drug user] can submit that after they’ve used. And what happens is the…the harm reduction agency, which we call collection sites, they just collect the sample, they ask the service user a few questions about that sample, you know, like what…what did you buy this drug as or get this drug as so it’s the expected drug. Was it associated with overdose or any adverse effects? What color is it? What texture is it? Like, why are you using the service kind of thing?”*

*(Alison, drug checking technician)*

#### 3.2.2. Legal and Political Landscapes

While there are exceptions and exemptions, possession of narcotics is illegal, which provides additional complications for this service. Participants described operating under the threat of prosecution for themselves and those seeking to access their services in the absence of legal protections. Here, Kelly, a drug checking technician, explains: “We’re at significant risk. There is a risk we will have a confrontation with law enforcement. There is a risk that’s real that we will be arrested if we are out on shift doing this and the right sort of storm of things happens around us.” Providers have consequently embraced mobile services, untethered to specific locations that may come under legal scrutiny, jeopardizing other services provided to the community. Drug checking at festivals and large-scale events, as seen in Europe and Canada, may also provide additional opportunities to entrench drug checking and increase its palatability.

Although California has a long history of supporting progressive drug policy, participants identified growing hostility from community members in the state to harm reduction and drug using populations as a growing problem, encapsulated by the experience of a drug checking program in the state:

*“We did not want to draw attention to the service, which is a novel service in the state and county, and the country to a certain extent, because the temperature or feelings around harm reduction in the city, there’s a lot of contention right now. And a lot of services are on blast. Providers on outreach and doing services in the community get a significant amount of harassment. And we’re starting also to get some of those harassment from police, which hasn’t happened since the ‘90s, which is very disturbing. But community members will come up, get in the face, physically assault, try and prevent services, film people getting medical services and getting supplies. So, we just want to be super mindful to try and protect our participants, to get our program well-established to the point where we feel safe before we make announcements.”*

*(Kelly)*

Some informants described how their respective drug checking programs were able to operate without the threat of prosecution due to exceptions associated with a grant or pilot program, or in Canada through government-sponsored exemptions, which offer lessons for how drug checking may be expanded in the USA. Still, even if these programs can operate without fear of prosecution, the possibility of public hostility to drug checking services may threaten their operations in other ways.

#### 3.2.3. An Innovative Enterprise

Overall, drug checking is characterized by innovation between drug checking providers and users, adapting to limitations in the legal and technological landscapes, and laying the framework for a system of confirmatory testing using multiple forms of technology to cross-check findings, which is reproducible across the USA and Canada:

*“And then, when you run one sample on many technologies, you get a really good idea of what’s actually in there, because some machines can’t detect this, some machines are really good at detecting this. Some machines can’t quantify, some machines can.”*

*(Jason)*

As mentioned above, participants thought that assuming fentanyl was present in street drugs and taking steps to mitigate overdose risk were essential, while they acknowledged both the psychological burden of this assumption and significant societal stigma about substance use. However, some were skeptical about the potential for drug checking alone as a mechanism for reducing overdose deaths, due both to the technology not being fully implemented in any location currently and to difficulties in scaling up the intervention and measuring its effect:

*“This is fundamentally one of the problems with our rationale for drug checking, is the assumption that we can behavior change our way out of risk or towards safety. And that is-I think it’s a faulty assumption […] one challenge, is that the technology is not fully dialed in yet, number one. And number two, the rationale that I keep hearing that people giving to funders for spending money on this, is to affect overdose. And one, there’s not a single study design that I can think of on the face of the planet that could actually correlate drug checking with a reduction in fatal overdose on a population level.”*

*(Sarah, harm reduction service provider)*

These participants took care to note that the increased focus on the health of people who use drugs and access these services would provide tangible benefits, including equity in access to health information that many take for granted and a means of reaching people where they are—a core tenet of harm-reduction-oriented services:

*“And so, for me, it’s a really big bodily autonomy, anti-drug war, health equity issue where it’s like in the same way that I can go into a store and look at the ingredients in whatever food that I’m going to eat. People should be able to know what are in the substances they’re using.”*

*(Kelly)*

### 3.3. Creation of Sustainable Programs

For drug checking programs to be sustainable and scalable, capacity building is sorely needed, and participants identified three areas most in need of development: staff education and training; financial investment; and policy changes to create a legal framework for drug checking services to operate.

#### 3.3.1. Staff Education and Training

While some informants described the FTIR machines as usable by the general population, most characterized the skill set needed to operate these machines as rarified and complex. Someone testing drug samples with FTIR needs the technical knowledge to operate the machine, a background in chemistry, and the analytical training to interpret the results. One participant acknowledged the dissonance between the reputation that drug checking machines have among harm reductionists and the reality of what it takes to use them in the field:

*“I think sometimes harm reduction people are like, ‘Oh, you just get one of those machines. And you just put it in the needle exchange, and then anybody can just kind of come up and test their drugs. And then you look at the computer, and it tells you what it is.’ And you’re like, ‘No. That’s actually not really totally how it works.”*

*(Jess, harm reduction service provider)*

Beyond technical and scientific knowledge, which are skilled but teachable competencies, providers experienced with using FTIR technology in the field said these skills alone are not enough. Knowledge and experience with drugs and drug culture came up repeatedly as critical qualifications to provide these services:

*“There’s that intuition of, you know, I know what heroin smells like. Or if I’m crushing up some crystal and there’s a certain texture about it, I’m like, ‘Oh. I can deduce X about this crystal,’ because I understand those things, it really has to do with understanding the baseline of your drug supply so you know what to look for when things look weird, it’s just challenging to find qualified people.”*

*(Kelly)*

This challenge of finding “qualified people”, that Kelly references, was echoed by other participants in this context. Sarah puts a finer point on it: The constellation of skills implies a single person with a background that includes familiarity with drugs and drug culture, as well as access to education and specialized training.

*“You need to know drug culture. And chemists are often academically arrogant, think that their way of…the knowledge that they bring to the table is the more important one, the more meaningful one. And so, that is the challenge with advanced technology, is we are in an actual crisis of trained folks to be able to operate them.”*

*(Sarah)*

While it is not contradictory for a person to have a background that includes both drug use and scientific training, what Sarah nods to here is that it is perhaps not common, which is a real factor when staffing.

#### 3.3.2. Financial Investment

Costliness of the FTIR machines came up frequently in interviews as a scalability and sustainability issue, particularly when paired with the delicacy of the machines. Participants reported that FTIR machines cost around USD 50,000, with participants noting that the accessibility presented by that price point broke down when scaling up the program for operating in the long term:

*“It’s an expensive machine. I think they go anywhere from, like, 30 to 60K, because of that, they’re not that scalable. I mean, we’re talking about, like, [organization] buying one and [University] has one now, I guess, but that means there’s one in L.A. for a city of 10 million. That’s just ridiculous; right? It…it’s…for this to be a useful intervention, we need 500 in my opinion; right? We need them to be everywhere, and we need these people to be able to access these services in a easy manner so they can actually use them.”*

*(Michael)*

Particularly for machines used in mobile outreach, durability has already been an issue:

*“It’s portable, but it’s not that portable. It’s apparently pretty vulnerable to humidity, wind, shaking, my more senior collaborators, understandably, don’t want me just taking it out in a van because, ‘This is really expensive. You’re going to break it.’“*

*(Amanda, researcher)*

Participants further noted that the cost of this technology could exacerbate inequities of access and care, where PWUDs in major metropolitan areas can access drug checking, but individuals living in smaller and more rural communities cannot:

*“Because of its cost and because of its high level of technical knowledge, that is going to kind of reinforce some resource access barriers that already exist. So, sure, if you are a very well-funded harm reduction program in [California], you can buy a $60,000 machine and hire three staff to run it.”*

*(Jess)*

Finally, many services were standalone interventions, not nested within larger sustained programming. These interventions were time-bound in their funding, and unless they received additional investment to keep them up and running, they would terminate at the end of the grant period:

*“We received five years of funding from the federal government. That money comes due-it expires at the end of January [2023] and we’re, you know, we’re the only drug checking service in the province of Ontario which is, you know, Canada’s most populated province, and, like, the government really has not supported us at all, and no one’s…I’m…I’m confident that someone will step up, but, like, at this point no one really is.”*

*(Alison)*

#### 3.3.3. Policy

Participants identified a central paradox when considering the sustainability and scalability of drug checking programs as they currently exist: Without special legislation or exemptions, these programs are illegal because the possession of these drugs is illegal, but legislation and exemptions that allow them to operate legally are often time-bound, location-specific, and precarious. The act of staffing and equipping a drug checking program is a major investment, but many of these programs are not built to be sustained past a certain point or to be scaled beyond certain tightly drawn boundaries:

*“Anywhere that it’s illegal to possess drugs, you’re going to have problems if there aren’t legal carve-outs for drug checking. A good question for me as I ponder: ‘Could I get arrested for supplying people with a mobile drug-checking device in certain U.S. states?’ That would be scary and not something that I would like to happen. It’s top of mind right now for me.”*

*(William)*

In addition to capacity building at the structural level with policy and financial investment, key informants offered recommendations for leveraging existing resources and networks to build capacity from the ground up:

*“We need a national drug checking Technical Assistance [TA] center. We need to have that ASAP, because [our program staff] and chemist spend a ton of time every week responding to requests from all over the country, like, ‘How do we set this technical thing up?’ or, ‘How do we reset the software to do this?’ And there is a very willing community of practice in the Alliance for Collaborative Drug Checking, but it’s time to have that be a national TA center.”*

*(Eli)*

Alison, who manages a drug checking program in Canada, gave a clear example of one way she contributed to this “community of practice”:

*“I’ve done seven of these exemption applications now and five for community health agencies, so I’ve actually created this really brief document that I call, like, pre-approve exempted processes for drug checking at community health agencies or collection sites, and I…we’ll share that with others who want to get drug checking programs up and running. And the federal government is aware that I’ve created this, and they, like, appear to be on board. And I’ve created them in a way that they’re high level enough that they should essentially be able to be adopted by anyone using any drug checking model.”*

*(Alison)*

### 3.4. Integrating Drug Checking into Other Health and Harm Reduction Services

Participants offered a range of perspectives on the relative merits of—and best strategies for—integrating drug checking services with other harm reduction or healthcare services. Some informants stated unambiguously that multiple forms of drug checking can and should be integrated with healthcare more broadly. Jess advocated for adding drug checking to existing harm reduction services, citing an overwhelming desire for the service among clients:

*“People are hungry for that information. I can’t imagine being at any harm reduction program where there’s drug checking available and people would be like, “Oh, no. I’m not interested.” People want to know what’s in their drug all the time.”*

*(Jess)*

For others, the opportunity presented by the appeal and low barrier to the entry of drug checking made service integration attractive. For those needing multiple health services but being disconnected from systems of care, drug checking could serve as an entry point:

*“I think engaging people in drug-checking services is a wonderful opportunity to connect them with healthcare, period-whether that’s healthcare maintenance, whether that’s bloodborne diseases, whether that’s a referral like an addiction medicine service. Absolutely, I think drug checking can be the conduit.”*

*(Melissa, harm reduction service provider)*

Other providers we interviewed were not so sure. Michael named the stigma associated with harm reduction within mainstream healthcare as a barrier to integration. He expressed doubt about the feasibility of integrating drug checking within a “mainstream institution,” while considering, too, the accessibility issues presented by asking people who use drugs to interact with an institution that stigmatizes them:

*“I think just the stigma, the institutional barriers would be enormously high to get them into like a mainstream institution. And then I think the use-the people who use drugs, it would be very challenging for them to even want to access these services. There’s a bunch of clinics that, I mean, they’ve never wanted our patients in their clinics, so don’t even try.”*

*(Michael)*

This issue, service integration increasing barriers for clients to access drug checking, was a central concern for multiple informants. Kelly worried that integrated services could push the boundary of becoming “transactional,” engendering a dynamic, real or perceived, in which drug checking is contingent upon participation in other healthcare activities or conversations. Similarly, Jason framed these cross-service interactions as potentially “crossing the line” and “asking a lot” of clients, particularly when talking to people who may have particularly fraught relationships with healthcare services:

*“But I think, as soon as you are asking people, ‘Hey, have you thought about checking out that abscess on your leg and going to the doctor?’ I think that it might be crossing the line. I think drug checking is best when it’s extremely low-barrier. And that generally means we’re not going to be asking a lot of you.”*

*(Jason)*

Other concerns included the feasibility of adding harm reduction and health services to existing drug checking programs. From a structural standpoint, service integration could mean raising the already high standard for program staff competencies:

*“I wouldn’t expect drug-checking technicians to feel comfortable or be trained well enough to tell someone, ‘Hey, your HIV test came back positive. You should go seek medical care.’ That’s a lot to expect someone to do.”*

*(Jason)*

While provider perspectives on whether and how to connect drug checking with other programming varied, one unifying concept ran through our dataset: relationships. Trusting relationships between clients and providers, more so than whether a program was formally integrated with other services or not, served as the primary mechanism for linking clients to further treatment or additional care.

Whether as part of a constellation of services or as a standalone program, drug checking gives providers the opportunity to build relationships with people who use drugs, many of whom are wary of interacting with health systems. This engagement begins with an informative service, in which a person can learn what they are putting in their body, but it does not necessarily end there. Melissa further explained how building relationships with clients both increases the likelihood that they will ask for referrals from a trusted provider if and when they are ready and can foster trust with mainstream healthcare systems as well. For many clients, those “bigger conversations” serve as a critical pathway to further treatment and engagement with systems of support:

*“We know from the evidence from other overdose prevention sites that people [who access harm reduction services] are more likely to enter treatment and detox. They’re more likely to engage around services like healthcare and housing. So, it’s just another place to really build relationships with people who currently don’t have those strong relationships with the system. It all starts with just inviting people to know the facts about what they’re putting in their bodies. I think that that service is just such a great service for people that it just opens the door, I think, for some trust and some gratitude that often lays the groundwork for those bigger conversations.”*

*(Melissa)*

## 4. Discussion

Providers presented a picture of the drug market as a moving target, characterized by rapid changes in localized drug supply content and associated rates of overdose. Drug checking services provide users with information about the risk of taking the respective drug compound tested and can influence drug-taking behaviors [65]. Indeed, as seen in the interviews conducted, drug checking services have been instrumental in monitoring and providing information to PWUDs about the presence of the highly potent synthetic opioid fentanyl, a product that has come to dominate the illicit drug market in a number of regions across North America [19,35,36,39,40,41,42,43]. Bearing witness to ever-changing drug markets, there is increasing concern over the presence of the veterinary tranquilizer xylazine within both the USA and Canada [66,67], and novel psychoactive substances of concern continue to emerge, requiring timely monitoring.

Overall, providers’ depictions of the illicit drug market highlight the need for drug checking as a key mechanism to reduce risks to PWUDs while emphasizing the ‘bootstrap’ nature of the implementation of these programs. In addition to providing individuals with vital information about potential drug risks, drug checking data can provide both formal and informal (word of mouth) warnings about contaminants and/or drug variants that may pose risks to the larger community [11,12,14,15]. In Canada, drug checking is increasingly used as a monitoring and surveillance tool, while other forms of drug supply surveillance have been suggested elsewhere [34,68]. It is notable how drug checking services emerged in the context of drug prohibition, creating an unregulated drug market where, outside of some law enforcement channels and limited public health surveillance, information about the drug supply is highly limited. Drug checking as a response to this sparse and sometimes guarded knowledge is not a monolith, but rather an amalgamation of established and novel services that seek to provide useful and actionable information about the drug supply. In short, drug checking services provide a key source of information for monitoring and acting upon changes in local drug markets.

While mindful of the promise that drug checking provides in terms of increasing individual- and community-level knowledge, drug checking providers recognized that drug checking is an imperfect enterprise, limited with respect to the available technology (and associated financial costs), the lack of combined technical and communication expertise requirements, and the legal landscape. With respect to the available technology (and reflecting the current literature), provider interviews highlighted that there is no single technology that can cope with ever-changing drug markets. While FTSs are cost-effective, can reliably identify fentanyl as a contaminant in other drugs, and are a useful component in a constellation of drug checking technologies, they identify only one compound among many and cannot offer quantitative data [39,49,69,70]. In addition, the utility of FTSs is reduced for those who prefer fentanyl or who are getting “fatigued” by needing to test frequently. By way of contrast, spectrometry provides information as to the overall content of the sample provided by drug users and can provide quantification as well, but the equipment is expensive, requires training to analyze the results, and the machines are limited with respect to their sensitivity (i.e., 5% by weight) and mobility [10,17,37,38,44,45,46,50]. To balance the pros and cons of each piece of technology, many programs are combining the greater sensitivity of FTSs (as well as immunoassay test strips for other drugs, e.g., benzodiazepines and xylazine) with the greater specificity of spectrometry in their drug checking algorithms.

Even if a single perfect technology was available, its costs, in terms of staffing and equipment, along with the need to provide integrated comprehensive health and social services for PWUDs remain challenging for most programs. Individual spectrometry machines were reported to cost approximately USD 50,000, but it is also clear that a single machine is not sufficient to cover a city the size of San Francisco (population~800,000), let alone Los Angeles (population~3,800,000). As such, it can be assumed that newly launched drug checking services would require significant funding support on a long-term basis and be in locations where PWUDs are best suited to access services [10,37,47,50]. Any upscaling efforts will require knowledge of how many machines (such as FTIR) would be needed to meet the requirements of a particular location (noting that some might need to be used in mobile units) and the upfront costs associated with these machines (and associated physical infrastructure), as well as training, operation, and maintenance costs.

Closely associated with technology and costs, providers pointed to a bottleneck or even crisis with respect to the staff needed to operate a successful drug checking program. Providers highlighted both the paucity of resources devoted to harm reduction programs generally, as well as the issue of training on and operating devices that require a high initial level of technical skill. Our study participants were divided on the appropriate level of technical skill as well as drug supply know-how needed to successfully operate and interpret the readout provided by a machine, such as an FTIR machine [64,71,72]. Finding appropriately trained individuals who are also knowledgeable about the drug supply and sympathetic to the issues faced by people who use drugs is likely to be challenging. Additionally, there is an urgent need, as participants noted, for developing training materials and technical support for the harm reduction organizations operating these programs. Asking that drug checking technicians, who are not deeply rooted in harm reduction practices, relay complicated information about drugs to clients is by no means easy, but participants were confident that training could be provided in this dual role of technical analyst and communicator. It is possible that a national training center and organizational hub could be leveraged as a resource for both technical support for drug checking technologies and communication expertise, as well to aggregate timely on-the-ground information about vicissitudes in the drug supply.

Underpinning these imperfections in technology and limitations in terms of scaling up service provision are the political and legal landscapes. Interviewees’ concerns regarding policy and policing reflect the considerable variation in the legal and policing context of drug checking within and outside of the USA [3,53,54,55,56]. The legal status of drug checking mentioned by some participants adds to the climate of uncertainty drug checking programs operate under. In Canada, for example, federal exemptions have been granted to implement drug checking services, but the process is time-consuming and fraught with logistical difficulties. Such uncertainty makes providers wary and, for structurally vulnerable communities, may reduce the likelihood of use [48,50,51,64].

Drug checking services suffer from an overall lack of official support at the state or federal levels, although several recent programs have launched in the USA that enjoy local support. Even in Canada, which has implemented drug checking for longer than the USA, many of these programs operate under temporary emergency authorizations, rather than enjoying guaranteed federal, state, and local support. On follow-up with a Canadian provider, funding for their program in Ontario is scheduled to run out at the end of March 2023, and no government or non-governmental entity has stepped up to provide long-term funding, leaving the staff uncertain in their job security and putting the community that this program serves in greater jeopardy. This speaks to the fundamental need for greater consistency in the policy and policing environment to enable providers some security that they will be allowed to provide this service above and beyond temporary exemption orders. Without such assurance, not only will PWUDs and providers be left in legal limbo, but it will remain difficult to find funding to pay for the necessary equipment and training investments without being able to plan for the future. Overall, if the legal status of drug checking remains as it is, drug checking services will continue to operate through temporary exemption orders and gray areas in the law, neither of which are compatible with meeting the long-term harm reduction objectives which encompass these initiatives [2,6,12,17,73].

Service providers showed considerable caution about how the impact of drug checking services should be measured, especially in terms of reducing overdose rates. Indeed, the question of whether providing drug checking findings to participants necessarily influences behavior change, directly reduces overdose rates, or improves uptake in substance use treatment programs cannot easily be answered at this time [13,15,42,74,75,76]. Nevertheless, the benefit in providing drug supply knowledge to PWUDs was one of the more dominant narratives emerging from the interviews. Knowing the content of their respective drug supply was seen as empowering, giving people with little say in the illicit drug supply a greater sense of control over their life and choices, including the choice to modify use, change drug sellers, and/or seek out further harm reduction services. As such, directly linking the success of drug checking services to overdose prevention may be unnecessarily reductive and counter-productive to the broad objectives of drug checking given the multiple indirect pathways by which these broad services may contribute to overdose prevention [1,10,11].

The success of drug checking services *may* be seen with individuals choosing to discard their drugs or reduce the frequency or amount taken to avoid likely harm [12,65,77], a change that could be life-saving but may be impossible to monitor and not always an option for those with few resources [41,48]. Other harm reducing behaviors include use of “tester shots” [78], carrying naloxone, not using alone, and using in the context of a supervised consumption space. However, even if behavioral change is not possible or not measurable, drug checking can still be empowering in linking individuals who use drugs to harm reduction services, including substance use treatment, HIV/HCV testing, housing assistance, supervised consumption sites, and medical care.

The access and engagement of PWUDs with other harm reduction services were viewed as key indicators of the success of drug checking programs. The collaborative nature and “meet people where they are at” sensibility of harm reduction programs were identified by many as particularly strong reasons to co-locate drug checking services within harm reduction programs, a finding not limited to participants directly employed in such organizations. Relationship building with participants was also a notable strength of harm reduction organizations identified in this research. In the case of those who have had negative, stigmatizing experiences with respect to policing, social attitudes, and/or when receiving healthcare services, drug checking in these venues can provide a foundation for engagement and trust building. From this point of trust, drug checking providers may be able to establish themselves (or their colleagues) as advocates for individuals wanting to access and navigate through health and social services that improve health outcomes.

Issuing public-facing warnings about drug supply variability remains challenging. At present, systems that provide warnings about ‘bad batches’, such as text-based warning systems seen in Baltimore, Maryland, USA [79] and in British Columbia, Canada [80], are generated from reported overdoses, including EMS data, rather than based on drug supply knowledge from drug checking. One potential measure of outcome success of warning programs would be a greater and more detailed ability to warn PWUDs about specific supply changes, including batch/brand warnings, appearance cues, and possible side effects and health risks. Early communication of drug checking findings across state and region may be another measure of success, while also acknowledging that local context is extremely important for understanding trends in drug contaminants. However, better monitoring through drug checking might also be instructive in building understanding about drug supply across cities, states, and regions. Another measure of success might be increases in the use of drug checking and/or associated harm reduction services. Other measures could include referrals from drug checking to HIV/HCV testing, first aid and wound care, and housing stabilization programs. Such information could be fed back to the drug user community as an indication of the benefits of drug checking and how drug checking can improve wellbeing. Moreover, they can be used as measures of success to demonstrate the utility of drug checking to policy makers and the general public who are sometimes hostile to drug checking and associated harm reduction measures [81].

Potential limitations of this study include its geographic restriction to North America. Drug checking is underway in many USA and Canadian localities, but its tenuous legal status makes recruitment of individuals working in drug checking particularly fraught. Snowball and targeted sampling of individuals working in a range of settings sought to address this limitation. Additionally, the lack of inclusion of the perspectives of PWUDs is another limitation. A companion manuscript from this study is in preparation that will explore perspectives of both providers and PWUDs on the feasibility of using drug checking services in the USA and report on the experiences some have already had with these services.

## 5. Conclusions

Drug checking, with all its creative variety and can-do mentality in an uncertain political environment, can be seen as a form of “bootstrap” public health. Various drug checking technologies have proven useful in providing valuable health information to people who use drugs, and programs have adapted these technologies to their needs. Despite the challenges posed, our participants described a burgeoning constellation of drug checking services that are well positioned to engage with and be embedded within existing harm reduction organizations, and potentially at trusted sites within or adjacent to the healthcare system. Drug checking programs on their own provide a short-term strategy to reduce the harms of an unstable and unsafe drug market. They operate, however, in the legal and policy landscapes which are unstable and uncertain, limiting their capacity to scale up operations to make PWUDs safer in the long term and make families and communities less vulnerable to the devastation of drug overdose morbidity and mortality. Additionally, there is an urgent need for a technical assistance center in North America to coordinate between programs and provide a vital conduit for information flow. A stable policy landscape, supportive of drug checking as part of harm reduction, would allow for financial investment in equipment, training, and service integration, which are much needed for this vulnerable community.

## Data Availability

The data are not publicly available due to privacy concerns about participant identities.

## References

[1] Barratt M.J., Measham F. (2022). What is drug checking, anyway?. Drugs Habits Soc. Policy.

[2] Brunt T. (2017). Drug Checking as a Harm Reduction Tool for Recreational Drug Users: Opportunities and Challenges.

[3] Davis C.S., Lieberman A.J., O’Kelley-Bangsberg M. (2022). Legality of drug checking equipment in the United States: A systematic legal analysis. Drug Alcohol Depend..

[4] Guirguis A., Gittins R., Schifano F. (2020). Piloting the UK’s first home-office-licensed pharmacist-led drug checking service at a community substance misuse service. Behav. Sci..

[5] Maghsoudi N., Tanguay J., Scarfone K., Rammohan I., Ziegler C., Werb D., Scheim A.I. (2021). Drug checking Services for People who use Drugs: A systematic review. Addiction.

[6] Masterton W., Falzon D., Burton G., Carver H., Wallace B., Aston E.V., Sumnall H., Measham F., Gittins R., Craik V. (2022). A realist review of how community-based drug checking services could be designed and implemented to promote engagement of people who use drugs. Int. J. Environ. Res. Public Health.

[7] Pu J., Ajisope T., Earlywine J. Drug Checking Programs in the United States and Internationally: Environmental Scan Summary 2021. https://aspe.hhs.gov/sites/default/files/documents/79e1975d5921d309ed924148ef019417/drug-checking-programs.pdf.

[8] Wallace B., van Roode T., Pagan F., Phillips P., Wagner H., Calder S., Aasen J., Pauly B., Hore D. (2020). What is needed for implementing drug checking services in the context of the overdose crisis? A qualitative study to explore perspectives of potential service users. Harm. Reduct. J..

[9] Wallace B., van Roode T., Pagan F., Hore D., Pauly B. (2021). The potential impacts of community drug checking within the overdose crisis: Qualitative study exploring the perspective of prospective service users. BMC Public Health.

[10] Giulini F., Keenan E., Killeen N., Ivers J.-H. (2022). A Systematized Review of Drug-checking and Related Considerations for Implementation as A Harm Reduction Intervention. J. Psychoact. Drugs.

[11] Measham F., Turnbull G. (2021). Intentions, actions and outcomes: A follow up survey on harm reduction practices after using an English festival drug checking service. Int. J. Drug Policy.

[12] Ivers J.-H., Killeen N., Keenan E. (2022). Drug use, harm-reduction practices and attitudes toward the utilisation of drug safety testing services in an Irish cohort of festival-goers. Ir. J. Med. Sci. (1971-).

[13] Palamar J.J., Fitzgerald N.D., Keyes K.M., Cottler L.B. (2021). Drug checking at dance festivals: A review with recommendations to increase generalizability of findings. Exp. Clin. Psychopharmacol..

[14] McCrae K., Tobias S., Tupper K., Arredondo J., Henry B., Mema S., Wood E., Ti L. (2019). Drug checking services at music festivals and events in a Canadian setting. Drug Alcohol Depend..

[15] Measham F., Simmons H. (2022). Who uses drug checking services? Assessing uptake and outcomes at English festivals in 2018. Drugs Habits Soc. Policy.

[16] Mema S.C., Sage C., Popoff S., Bridgeman J., Taylor D., Corneil T. (2018). Expanding harm reduction to include fentanyl urine testing: Results from a pilot in rural British Columbia. Harm. Reduct. J..

[17] Harper L., Powell J., Pijl E.M. (2017). An overview of forensic drug testing methods and their suitability for harm reduction point-of-care services. Harm. Reduct. J..

[18] Snowdon J., Choi N. (2022). Unanticipated Changes in Drug Overdose Death Rates in Canada During the Opioid Crisis. Int. J. Ment. Health Addict..

[19] Ciccarone D. (2021). The rise of illicit fentanyls, stimulants and the fourth wave of the opioid overdose crisis. Curr. Opin. Psychiatry.

[20] Compton W.M., Einstein E.B., Jones C.M. (2022). Exponential increases in drug overdose: Implications for epidemiology and research. Int. J. Drug Policy.

[21] Ciccarone D. (2019). The triple wave epidemic: Supply and demand drivers of the US opioid overdose crisis. Int. J. Drug Policy.

[22] Jalal H., Burke D.S. (2022). Exponential growth of drug overdose poisoning and opportunities for intervention. Addiction.

[23] Friedman J., Akre S. (2021). COVID-19 and the drug overdose crisis: Uncovering the deadliest months in the United States, January–July 2020. Am. J. Public Health.

[24] Friedman J.R., Hansen H. (2022). Evaluation of increases in drug overdose mortality rates in the US by race and ethnicity before and during the COVID-19 pandemic. JAMA Psychiatry.

[25] National Institute on Drug Abuse (2023). Drug Overdose Death Rates. https://nida.nih.gov/research-topics/trends-statistics/overdose-death-rates.

[26] Jalal H., Buchanich J.M., Roberts M.S., Balmert L.C., Zhang K., Burke D.S. (2018). Changing dynamics of the drug overdose epidemic in the United States from 1979 through 2016. Science.

[27] Friedman J., Beletsky L., Jordan A. (2022). Surging racial disparities in the US overdose crisis. Am. J. Psychiatry.

[28] Canadian Centre on Substance Use (CCSA) (2015). Deaths Involving Fentanyl in Canada, 2009–2014 (CCENDU Bulletin).

[29] Belzak L., Halverson J. (2018). Evidence synthesis—The opioid crisis in Canada: A national perspective. Health Promot. Chronic Dis. Prev. Can. Res. Policya Pract..

[30] Government of Canada Opioid- and Stimulant-Related Harms—Canada.ca. https://health-infobase.canada.ca/substance-related-harms/opioids-stimulants/.

[31] Ciccarone D. (2017). Fentanyl in the US heroin supply: A rapidly changing risk environment. Int. J. Drug Policy.

[32] Ciccarone D., Ondocsin J., Mars S.G. (2017). Heroin uncertainties: Exploring users’ perceptions of fentanyl-adulterated and-substituted ‘heroin’. Int. J. Drug Policy.

[33] Palamar J.J., Ciccarone D., Rutherford C., Keyes K.M., Carr T.H., Cottler L.B. (2022). Trends in seizures of powders and pills containing illicit fentanyl in the United States, 2018 through 2021. Drug Alcohol Depend..

[34] Rosenblum D., Unick J., Ciccarone D. (2020). The Rapidly Changing US Illicit Drug Market and the Potential for an Improved Early Warning System: Evidence from Ohio Drug Crime Labs. Drug Alcohol Depend..

[35] Donroe J.H., Socias M.E., Marshall B.D. (2018). The deepening opioid crisis in North America: Historical context and current solutions. Curr. Addict. Rep..

[36] O’Donnell J., Tanz L.J., Gladden R.M., Davis N.L., Bitting J. (2021). Trends in and characteristics of drug overdose deaths involving illicitly manufactured fentanyls—United States, 2019–2020. Morb. Mortal. Wkly. Rep..

[37] Green T.C., Park J.N., Gilbert M., McKenzie M., Struth E., Lucas R., Clarke W., Sherman S.G. (2020). An assessment of the limits of detection, sensitivity and specificity of three devices for public health-based drug checking of fentanyl in street-acquired samples. Int. J. Drug Policy.

[38] Palamar J.J., Salomone A., Barratt M.J. (2020). Drug checking to detect fentanyl and new psychoactive substances. Curr. Opin. Psychiatry.

[39] Park J.N., Frankel S., Morris M., Dieni O., Fahey-Morrison L., Luta M., Hunt D., Long J., Sherman S.G. (2021). Evaluation of fentanyl test strip distribution in two Mid-Atlantic syringe services programs. Int. J. Drug Policy.

[40] Beaulieu T., Hayashi K., Nosova E., Milloy M.J., DeBeck K., Wood E., Kerr T., Ti L. (2020). Effect of witnessing an overdose on the use of drug checking services among people who use illicit drugs in Vancouver, Canada. Am. J. Drug Alcohol Abus..

[41] Karamouzian M., Dohoo C., Forsting S., McNeil R., Kerr T., Lysyshyn M. (2018). Evaluation of a fentanyl drug checking service for clients of a supervised injection facility, Vancouver, Canada. Harm. Reduct. J..

[42] Rouhani S., Park J.N., Morales K.B., Green T.C., Sherman S.G. (2019). Harm reduction measures employed by people using opioids with suspected fentanyl exposure in Boston, Baltimore, and Providence. Harm. Reduct. J..

[43] Ramsay M., Gozdzialski L., Larnder A., Wallace B., Hore D. (2021). Fentanyl quantification using portable infrared absorption spectroscopy. A framework for community drug checking. Vib. Spectrosc..

[44] Borden S.A., Saatchi A., Vandergrift G.W., Palaty J., Lysyshyn M., Gill C.G. (2022). A new quantitative drug checking technology for harm reduction: Pilot study in Vancouver, Canada using paper spray mass spectrometry. Drug Alcohol Rev..

[45] Gozdzialski L., Aasen J., Larnder A., Ramsay M., Borden S.A., Saatchi A., Gill C.G., Wallace B., Hore D.K. (2021). Portable gas chromatography–mass spectrometry in drug checking: Detection of carfentanil and etizolam in expected opioid samples. Int. J. Drug Policy.

[46] Laxton J.-C., Monaghan J., Wallace B., Hore D., Wang N., Gill C.G. (2023). Evaluation and improvement of a miniature mass spectrometry system for quantitative harm reduction drug checking. Int. J. Mass Spectrom..

[47] Karch L., Tobias S., Schmidt C., Doe-Simkins M., Carter N., Salisbury-Afshar E., Carlberg-Racich S. (2021). Results from a mobile drug checking pilot program using three technologies in Chicago, IL, USA. Drug Alcohol Depend..

[48] Bardwell G., Boyd J., Tupper K.W., Kerr T. (2019). “We don’t got that kind of time, man. We’re trying to get high!”: Exploring potential use of drug checking technologies among structurally vulnerable people who use drugs. Int. J. Drug Policy.

[49] Krieger M.S., Yedinak J.L., Buxton J.A., Lysyshyn M., Bernstein E., Rich J.D., Green T.C., Hadland S.E., Marshall B.D. (2018). High willingness to use rapid fentanyl test strips among young adults who use drugs. Harm. Reduct. J..

[50] Carroll J.J., Mackin S., Schmidt C., McKenzie M., Green T.C. (2022). The Bronze Age of drug checking: Barriers and facilitators to implementing advanced drug checking amidst police violence and COVID-19. Harm. Reduct. J..

[51] Rammohan I., Bouck Z., Fusigboye S., Bowles J., McDonald K., Maghsoudi N., Scheim A., Werb D. (2022). Drug checking use and interest among people who inject drugs in Toronto, Canada. Int. J. Drug Policy.

[52] Scott I.A., Scott R.J. (2020). Pill testing at music festivals: Is it evidence-based harm reduction?. Intern. Med. J..

[53] Bacon M. (2022). Desistance from criminalisation: Police culture and new directions in drugs policing. Polic. Soc..

[54] Kammersgaard T. (2019). Harm reduction policing: From drug law enforcement to protection. Contemp. Drug Probl..

[55] Watson T.M., Bayoumi A.M., Hopkins S., Wright A., Naraine R., Khorasheh T., Challacombe L., Strike C. (2018). Creating and sustaining cooperative relationships between supervised injection services and police: A qualitative interview study of international stakeholders. Int. J. Drug Policy.

[56] Khorasheh T., Naraine R., Watson T.M., Wright A., Kallio N., Strike C. (2019). A scoping review of harm reduction training for police officers. Drug Alcohol Rev..

[57] Maghsoudi N., McDonald K., Stefan C., Beriault D.R., Mason K., Barnaby L., Altenberg J., MacDonald R.D., Caldwell J., Nisenbaum R. (2020). Evaluating networked drug checking services in Toronto, Ontario: Study protocol and rationale. Harm. Reduct. J..

[58] Bardwell G., Strike C., Mitra S., Scheim A., Barnaby L., Altenberg J., Kerr T. (2020). “That’s a double-edged sword”: Exploring the integration of supervised consumption services within community health centres in Toronto, Canada. Health Place.

[59] Scheim A., Werb D. (2018). Integrating supervised consumption into a continuum of care for people who use drugs. CMAJ.

[60] Nassau T., Kolla G., Mason K., Hopkins S., Tookey P., McLean E., Werb D., Scheim A. (2022). Service utilization patterns and characteristics among clients of integrated supervised consumption sites in Toronto, Canada. Harm. Reduct. J..

[61] Greenwald Z.R., Bouck Z., McLean E., Mason K., Lettner B., Broad J., Dodd Z., Nassau T., Scheim A., Werb D. (2022). Integrated supervised consumption services and hepatitis C testing and treatment among people who inject drugs in Toronto, Canada: A cross-sectional analysis. J. Viral Hepat..

[62] Glick J.L., Christensen T., Park J.N., McKenzie M., Green T.C., Sherman S.G. (2019). Stakeholder perspectives on implementing fentanyl drug checking: Results from a multi-site study. Drug Alcohol Depend..

[63] Miles M.B., Huberman A.M. (1994). Qualitative Data Analysis: An Expanded Sourcebook.

[64] McCrae K., Tobias S., Grant C., Lysyshyn M., Laing R., Wood E., Ti L. (2020). Assessing the limit of detection of Fourier-transform infrared spectroscopy and immunoassay strips for fentanyl in a real-world setting. Drug Alcohol Rev..

[65] Peiper N.C., Clarke S.D., Vincent L.B., Ciccarone D., Kral A.H., Zibbell J.E. (2019). Fentanyl test strips as an opioid overdose prevention strategy: Findings from a syringe services program in the Southeastern United States. Int. J. Drug Policy.

[66] Friedman J., Montero F., Bourgois P., Wahbi R., Dye D., Goodman-Meza D., Shover C. (2022). Xylazine spreads across the US: A growing component of the increasingly synthetic and polysubstance overdose crisis. Drug Alcohol Depend..

[67] Bowles J.M., McDonald K., Maghsoudi N., Thompson H., Stefan C., Beriault D.R., Delaney S., Wong E., Werb D. (2021). Xylazine detected in unregulated opioids and drug administration equipment in Toronto, Canada: Clinical and social implications. Harm. Reduct. J..

[68] Centre on Drug Policy Evaluation Toronto’s Drug Checking Service. https://drugchecking.cdpe.org/.

[69] Bergh M.S.-S., Øiestad Å.M.L., Baumann M.H., Bogen I.L. (2021). Selectivity and sensitivity of urine fentanyl test strips to detect fentanyl analogues in illicit drugs. Int. J. Drug Policy.

[70] Laing M.K., Tupper K.W., Fairbairn N. (2018). Drug checking as a potential strategic overdose response in the fentanyl era. Int. J. Drug Policy.

[71] Cirrincione M., Saladini B., Brighenti V., Salamone S., Mandrioli R., Pollastro F., Pellati F., Protti M., Mercolini L. (2021). Discriminating different Cannabis sativa L. chemotypes using attenuated total reflectance-infrared (ATR-FTIR) spectroscopy: A proof of concept. J. Pharm. Biomed. Anal..

[72] Ti L., Tobias S., Lysyshyn M., Laing R., Nosova E., Choi J., Arredondo J., McCrae K., Tupper K., Wood E. (2020). Detecting fentanyl using point-of-care drug checking technologies: A validation study. Drug Alcohol Depend..

[73] Bolinski R.S., Walters S., Salisbury-Afshar E., Ouellet L.J., Jenkins W.D., Almirol E., Van Ham B., Fletcher S., Johnson C., Schneider J.A. (2022). The Impact of the COVID-19 Pandemic on Drug Use Behaviors, Fentanyl Exposure, and Harm Reduction Service Support among People Who Use Drugs in Rural Settings. Int. J. Environ. Res. Public Health.

[74] Kennedy M.C., Scheim A., Rachlis B., Mitra S., Bardwell G., Rourke S., Kerr T. (2018). Willingness to use drug checking within future supervised injection services among people who inject drugs in a mid-sized Canadian city. Drug Alcohol Depend..

[75] Measham F.C. (2019). Drug safety testing, disposals and dealing in an English field: Exploring the operational and behavioural outcomes of the UK’s first onsite ‘drug checking’service. Int. J. Drug Policy.

[76] Trayner K.M., Palmateer N.E., Hutchinson S.J., Goldberg D.J., Shepherd S.J., Gunson R.N., Tweed E.J., Priyadarshi S., Sumnall H., Atkinson A. (2021). High willingness to use drug consumption rooms among people who inject drugs in Scotland: Findings from a national bio-behavioural survey among people who inject drugs. Int. J. Drug Policy.

[77] Betzler F., Helbig J., Viohl L., Ernst F., Roediger L., Gutwinski S., Ströhle A., Köhler S. (2021). Drug Checking and Its Potential Impact on Substance Use. Eur. Addict. Res..

[78] Mars S.G., Ondocsin J., Ciccarone D. (2018). Toots, tastes and tester shots: User accounts of drug sampling methods for gauging heroin potency. Harm. Reduct. J..

[79] Bad Batch Alert. http://www.badbatchalert.com/.

[80] (2023). British Columbia Ministry of Mental Health and Addictions Real-Time Drug Alert and Response. https://www.wellbeing.gov.bc.ca/resource/real-time-drug-alert-and-response.

[81] Barry C.L., Sherman S.G., Stone E., Kennedy-Hendricks A., Niederdeppe J., Linden S., McGinty E.E. (2019). Arguments supporting and opposing legalization of safe consumption sites in the US. Int. J. Drug Policy.

